# Association Between Biochemical, Inflammatory, Oxidative Stress and DNA Methylation Biomarkers with Perceived Stress in Mexican Individuals

**DOI:** 10.3390/biom16030405

**Published:** 2026-03-10

**Authors:** Heriberto Jacobo-Cuevas, Laura González-López, Saúl Ramírez-de-Los-Santos, Ana Míriam Saldaña-Cruz, Juan Manuel Ponce-Guarneros, Norma Alejandra Rodríguez-Jimenez, Aniel Jessica Leticia Brambila-Tapia

**Affiliations:** 1 Group of Assessment of Prognosis Biomarkers in Autoimmune Disorders, Centro Universitario de Ciencias de la Salud (CUCS), Universidad de Guadalajara, Guadalajara 44340, Mexico; heriberto.jcuevas@academicos.udg.mx (H.J.-C.); ldelcarmen.gonzalez@academicos.udg.mx (L.G.-L.); norma.rodriguezj@academicos.udg.mx (N.A.R.-J.); 2 Programa de Postdoctorado, Departamento de Psicología Básica, Centro Universitario de Ciencias de la Salud (CUCS), Universidad de Guadalajara, Guadalajara 44340, Mexico; 3 Instituto de Terapéutica Experimental y Clínica (INTEC), Departamento de Fisiología, Centro Universitario de Ciencias de la Salud (CUCS), Universidad de Guadalajara, Guadalajara 44340, Mexico; ana.saldanac@academicos.udg.mx (A.M.S.-C.); juan.ponce4091@academicos.udg.mx (J.M.P.-G.); 4 Departamento de Psicología Básica, Centro Universitario de Ciencias de la Salud (CUCS), Universidad de Guadalajara, Guadalajara 44340, Mexico; saul.rdelossantos@academicos.udg.mx; 5 Instituto de Investigación en Ciencias Biomédicas (IICB), Centro Universitario de Ciencias de la Salud (CUCS), Universidad de Guadalajara, Guadalajara 44340, Mexico

**Keywords:** perceived stress, oxidative stress, inflammation, DNA global methylation, biomarkers

## Abstract

Stress is increasingly recognized as a complex, multidimensional phenomenon shaped by interacting biological, psychological, and social factors, and it has been linked to numerous physical conditions. Several inflammatory and oxidative stress markers have been correlated with perceived stress. However, the combined association of biochemical variables, inflammatory and oxidative stress biomarkers, and DNA methylation with perceived stress has not yet been examined in the Mexican population. Therefore, the objective of this study was to determine such an association in a sample of non-representative Mexican adult individuals. A total of 157 individuals were included, of whom 83 (53%) were women. Women showed higher values of stress than men. In the bivariate correlations, perceived stress correlated negatively with sleep quality, age, total cholesterol, monthly earnings and waist-to-hip ratio and positively with morbidity count, leucocytes and platelets. In the multivariable analyses, additional variables were associated with perceived stress, including a positive correlation with IL-1β in the total sample, a positive correlation with 8-isoprostane in the women’s sample, and a negative correlation with this molecule in the men’s sample. Similarly, perceived stress correlated positively with DNA global methylation in the men’s sample and negatively with this variable in the women’s sample. In conclusion, perceived stress showed correlations with many variables, including sociodemographic and behavioral ones, such as sex, age and sleep quality; biochemical variables, including serum lipids, platelets and leukocytes; and inflammation (IL-1β), oxidative stress (8-isoprostane) and DNA methylation (global DNA methylation) biomarkers, some of them showing opposite correlations in each sex.

## 1. Introduction

Stress has been classically defined as a nonspecific physiological response of an organism to any demand that disrupts homeostasis. Hans Selye (1936) first described it as “the non-specific response of the body to any demand,” introducing the concept of general adaptation syndrome, which comprises the alarm, resistance, and exhaustion phases [1]. Later, Lazarus and Folkman (1984) expanded the concept to the psychological domain, defining stress as a transactional relationship between the individual and the environment, in which the person appraises environmental demands as exceeding their coping resources, thereby threatening well-being [2]. From a contemporary neuroendocrine perspective, McEwen and Stellar (1993) [3] introduced the concepts of allostasis and allostatic load, describing stress as an adaptive process that maintains physiological stability through change. However, when stress is chronic or excessive, it leads to the dysregulation of neuroendocrine, immune, and metabolic axes [3]. Overall, stress can be understood as a multidimensional process involving biological, psychological, and social components, whose persistence or dysregulation contributes to the onset, progression, and prognosis of numerous physical conditions and the development of pathophysiological changes in the cardiovascular, metabolic, immune, and neuroendocrine systems, as well as to the emergence of somatic symptoms [4]. Elevated perceived stress has been consistently associated with cardiovascular disease (CVD) through mechanisms involving increased blood pressure, endothelial dysfunction, and systemic inflammation [5,6]. High-stress individuals show higher circulating levels of IL-6, CRP, and fibrinogen, which are predictive of myocardial infarction and atherosclerotic progression [7]. Similarly, stress-related dysregulation of the hypothalamus–pituitary–adrenal (HPA) axis and sympathetic activity contributes to metabolic disturbances, including insulin resistance, abdominal obesity, and type 2 diabetes mellitus [8,9]. Chronic stress is also associated with impaired immune responses, predisposing individuals to infectious diseases and slower wound healing [10]. Furthermore, stress is closely linked to psychosomatic and functional disorders, such as irritable bowel syndrome (IBS), fibromyalgia, chronic fatigue syndrome, and tension-type headaches, where stress-mediated neuroimmune interactions exacerbate symptom severity [11,12,13]. At the cellular level, chronic stress has been correlated with shorter telomere length, increased oxidative damage, and accelerated biological aging, highlighting its systemic impact beyond psychological well-being [14]. In summary, stress functions as a psychobiological bridge connecting psychosocial experiences with physical disease processes, mediated by chronic inflammation, oxidative imbalance, and maladaptive epigenetic and neuroendocrine responses [15].

A growing body of evidence has demonstrated that perceived stress—as quantified by validated scales such as the perceived stress scale (PSS) [16,17]—is associated with measurable alterations in biochemical and molecular markers, reflecting inflammatory activity, oxidative balance, and DNA methylation status [18,19,20]. Cross-sectional and longitudinal studies have shown that higher PSS scores correlate with elevated C-reactive protein (CRP), interleukin-6 (IL-6), and tumor necrosis factor-alpha (TNF-α) levels, reflecting low-grade systemic inflammation. These associations often persist after adjustment for confounding factors such as age, sex, BMI, and depressive symptoms, suggesting an independent contribution of stress-related pathways to immune dysregulation [21,22,23]. Similarly, perceived stress has been linked to increased oxidative stress markers, including 8-isoprostane (8-iso-PGF_2_α) and 8-hydroxy-2′-deoxyguanosine (8-OHdG), as well as reduced total antioxidant capacity [24,25]. Chronic psychosocial stress appears to shift the redox equilibrium toward a pro-oxidant state, promoting oxidative damage to cellular macromolecules and mitochondrial dysfunction [26]. Recent findings also indicate that perceived stress is related to epigenetic modifications, such as alterations in global DNA methylation (e.g., LINE-1 and Alu elements) and methylation of stress-regulatory genes (NR3C1, FKBP5, and BDNF) [27,28,29,30]. These epigenetic changes may mediate the biological embedding of stress, influencing gene expression patterns associated with inflammation, oxidative stress, and aging processes [31,32,33]. Collectively, these data underscore the role of perceived stress as a psychobiological construct with measurable effects on immune, oxidative, and epigenetic homeostasis, supporting its inclusion in integrative biomarker research frameworks.

Although numerous studies have examined the relationship between perceived stress and biomarkers of oxidative stress, inflammation, and epigenetic regulation, to date, no research has specifically analyzed the association between perceived stress and global DNA methylation. Consequently, there are also no studies evaluating this relationship while adjusting for biochemical, oxidative stress, and inflammatory biomarkers in a population sample stratified by sex.

Therefore, the aim of this study was to evaluate associations of a comprehensive set of personal, biochemical, inflammatory and oxidative stress biomarkers with perceived stress in Mexican individuals.

## 2. Subjects and Methods

### 2.1. Ethical Considerations and Study Population

This study complied with the Declaration of Helsinki and was approved by the Research and Ethics Committee of the University Center for Health Sciences (approval CI-06123; 25 September 2024). All participants provided written informed consent.

### 2.2. Subjects

Eligible participants met the following criteria: (a) 18–60 years of age; (b) not pregnant; and (c) no genetic relationship to any other participant (e.g., siblings or cousins). The exclusion criterion was missing data for any study variable.

Study design: This is an observational, cross-sectional study.

### 2.3. Procedures

Recruitment was conducted over two months via announcements on social media (e.g., WhatsApp, version 2024) and printed brochures. Students and staff from the University Center for Health Sciences were also approached in person. Eligibility was verified by the research team. Individuals who consented were scheduled in a University of Guadalajara computer room, where they provided written informed consent and completed an electronic questionnaire (Google Forms) collecting personal information. Before the questionnaire, anthropometric measures were performed, and blood pressure was determined.

### 2.4. Personal Variables

The personal and sociodemographic variables included sex; age; partnership status; parenthood; educational attainment; monthly earnings; employment status; daily minutes of physical activity; daily hours of leisure; and the frequency of alcohol, tobacco, and illicit drug use. Substance-use frequency was rated on a five-point scale from “never” to “four or more times per week”. Self-reported morbidity over the previous six months covered 29 conditions: see Supplementary Table S1.

We also recorded the count of different medications taken each day (“daily drug intake”) and weekly dietary supplement use, the latter through a question that comprises five options, from 0 (never) to 4 (daily).

### 2.5. Sleep Quality Assessment

Sleep quality was evaluated through the second component of the OVIEDO sleep questionnaire, which comprises five items, each rated on a scale from 0 (poor quality) to 4 (excellent quality) [34].

### 2.6. Psychological Assessments

To measure perceived stress, we used the perceived stress scale (PSS-10) [16,35]. Higher scores indicate greater levels of perceived stress. Depressive symptoms were assessed with the patient health questionnaire (PHQ-9) [36]. This scale was used only to compare the correlation between depressive symptoms and serum lipids.

### 2.7. Venous Blood Sampling

Early-morning fasting venous blood (≥8 h) was collected from all participants via venipuncture by trained personnel on the research team. The samples were then subjected to biochemical analyses.

### 2.8. Measurement of Biochemical Variables

Venous blood from all participants was analyzed to quantify: (1) complete blood count (hemoglobin, platelets, leukocytes, and differential counts); (2) total cholesterol (TC) and triglycerides; and (3) basic blood chemistry (glucose and urea). Complete blood counts were measured by electronic impedance (Model HORIBA ABX Micros ES 60 Hematology Analyzer, Horiba Ltd., Montpellier, France), and all chemistry assays were performed by colorimetry (Model H-100 Automated Clinical Chemistry Analyzer, HLAB Supply Ltd., Denver, CO, USA).

Anthropometric measurements were obtained following standardized procedures. Body mass index (BMI) was calculated as weight in kilograms divided by height in meters squared (kg/m^2^). The waist-to-hip ratio (WHR) was determined by measuring waist and hip circumferences and calculating their ratio. Systolic and diastolic blood pressure were measured using an Omron upper-arm blood pressure monitor (Model HEM-7320, OMRON Healthcare Co., Ltd., Kyoto, Japan) under resting conditions. To verify measurement accuracy, readings obtained with this device were cross-checked against those measured using a standard manual blood pressure cuff in the first and last participants enrolled in the study.

### 2.9. Serum Inflammatory and Oxidative-Stress Biomarkers

Serum biomarker concentrations were determined by ELISA according to each kit’s manufacturer instructions. Aliquots were stored at −80 °C until analysis. The following ELISA kits from MyBioSource (MyBioSource, Inc., San Diego, CA, USA) were used: TNF-α (MBS175820), IL-8 (MBS763092), IL-6 (MBS021993), IL-1β (MBS263843), IL-10 (MBS764410), 8-isoprostane (MBS3802509), and 8-hydroxy-2′-deoxyguanosine (MBS267161).

### 2.10. DNA Extraction

Genomic DNA was isolated from peripheral blood leukocytes using a modified Miller protocol with CTAB/DTAB. DNA yield (A260) and purity (A260/A280) were assessed on a NanoDrop spectrophotometer (NanoDrop Technologies Inc., Delaware, TX, USA). Extracts were then diluted in Tris–EDTA (TE) buffer to a final concentration of 100 ng/µL.

### 2.11. ELISA-Based Quantification of Global DNA Methylation

Global DNA methylation was evaluated in duplicate using 100 ng of leukocyte DNA. 5-methylcytosine (5-mC) was detected with capture and detection antibodies and quantified by a colorimetric assay against a standard curve, reading absorbance at 450 nm on a Multiskan™ FC microplate spectrophotometer (Thermo Fisher Scientific, Waltham, MA, USA). Optical density was directly proportional to methylation levels. Quantification followed the manufacturer’s protocol using the MethylFlash™ Methylated DNA Quantification Kit (Epigentek, P-1030; Farmingdale, NY, USA).

### 2.12. Statistical Analysis

Continuous variables were summarized as mean ± SD for approximately normal distributions and as median (range) for non-normal distributions; categorical variables were presented as counts and percentages. Sex differences in sociodemographic variables were tested with chi-square (categorical) and Student’s *t*-test or the Mann–Whitney U test (continuous, according to distribution). Associations between perceived stress with each personal, biochemical and anthropometric measure—as well as inflammatory and oxidative-stress biomarkers and global DNA methylation—were examined using Pearson or Spearman correlations, as appropriate. To identify variables independently associated with perceived stress, we fitted a multiple linear regression using a stepwise procedure. The dependent variable (perceived stress) was continuous and normally distributed; candidate predictors included continuous, dichotomous, and ordinal variables. Analyses were performed in the full sample and stratified by sex to account for potential confounding; reported *p*-values are adjusted within the multivariable model. All analyses were conducted using SPSS v31; statistical significance was set at *p* < 0.05 (two-tailed).

## 3. Results

A total of 176 individuals consented to participate. Nineteen were excluded for incomplete assessments, leaving 157 participants for analysis. Participant characteristics and sex comparisons are summarized in Table 1. Of those included, 83 (52.8%) were women; the median age was 24 years (range 18–58). Approximately 67% were employed, and 68.8% reported monthly earnings in the lower- to upper-middle brackets (7474–17,308 MXN). Women reported lower monthly earnings and lower educational attainment than men. Regarding health-related variables, women reported a greater morbidity count, whereas men showed a higher high-frequency of alcohol use and greater illicit substance use. Women also exhibited higher levels of perceived stress (1.6 vs. 1.4; *p* = 0.048) than men. Among biochemical measures, several sex differences reached significance. Women showed higher platelet counts than men (236.5 ± 48.9 vs. 209.4 ± 40.7 × 10^3^/µL; *p* < 0.001). In contrast, men had higher hemoglobin (median 14.5 vs. 12.7 g/dL; *p* < 0.001) and higher fasting glucose (median 92.3 vs. 83.8 mg/dL; *p* < 0.001). Global DNA methylation was also modestly higher in men (median 0.5% vs. 0.4%; *p* = 0.045). In relation to anthropometrics, BMI was comparable between sexes, whereas both systolic and diastolic blood pressure were higher in men (*p* < 0.001 and *p* = 0.025, respectively). Men also had a greater WHR—median 0.87 vs. 0.78 in women (*p* < 0.001).

Table 2 summarizes the bivariate correlations between perceived stress and study variables that reached statistical significance in the full sample or in at least one sex-specific subgroup. In the total sample, higher perceived stress showed a moderate negative correlation with sleep quality; low negative correlations with age and total cholesterol; and very low negative correlations with monthly earnings, waist-to-hip ratio, and triglycerides. In addition, perceived stress showed a weak positive correlation with morbidity count, leukocyte and platelet counts.

In sex-stratified analyses, among women, perceived stress showed a moderate negative correlation with sleep quality, weak negative correlations with age and total cholesterol, and a very low negative correlation trend toward 8-hydroxy-2′-deoxyguanosine; in addition, perceived stress correlated positively (very low) with platelet count and illicit drug use and showed a trend toward a positive correlation with morbidity count. Among men, perceived stress was modestly inversely related to sleep quality, monthly earnings, and triglycerides and showed a trend toward a positive association with leukocytes. Additionally, we examined the correlation between TC and triglycerides with depressive symptoms (PHQ-9) in the global sample, segmented by sex; the correlation between depressive symptoms with TC and triglycerides in the total sample was rho = −0.334, *p* < 0.01, and rho = −0.275. *p* < 0.01, respectively. In women, rho = −0.302, *p* < 0.01, and rho = −0.160 (*p* > 0.05), and in men, rho = −0.321, *p* < 0.01, and rho = −0.381, *p* < 0.01.

**Figure 1:**
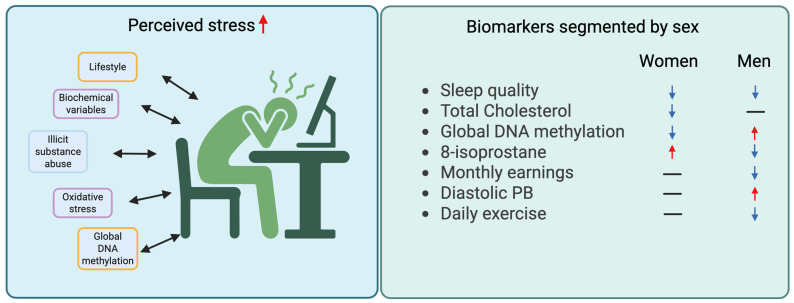
Relationships between identified biomarkers associated with perceived stress. The figure represents the group of variables associated with perceived stress (**left side**), with bidirectional arrows indicating that the causal relationship can be bilateral, and the relationships between identified biomarkers associated with perceived stress in the sex-segmented multivariable analyses (**right side**). Arrows indicate the direction of association (↑ positive association; ↓ negative association), while horizontal bars (—) indicate non-significant associations.

In the full multivariable model, perceived stress scores were positively associated with employment (employed), platelet count, IL-1β, and morbidity count and inversely correlated with sleep quality, total cholesterol, age, and daily physical activity. In sex-stratified models, among women, perceived stress was positively correlated with 8-isoprostane and inversely correlated with sleep quality, total cholesterol, and global DNA methylation. Among men, perceived stress was positively correlated with global DNA methylation, DBP, and erythrocyte count and inversely correlated with sleep quality, 8-isoprostane, monthly earnings, WHR, and daily physical activity (Table 3). These findings are presented in the Figure 1, summarizing the key differences between variables identified in the sex-stratified analysis.

The multivariable regression models showed a satisfactory fit. In the total sample, R = 0.592, and R^2^ = 0.350, indicating that predictors accounted for 35.0% of the variance in perceived stress. Among women, R = 0.626 with R^2^ = 0.392 (39.2% of variance explained), and among men, R = 0.668 with R^2^ = 0.446 (44.6% explained).

## 4. Discussion

In the bivariate analysis, we found that the female group exhibited a greater number of statistically significant associations with perceived stress. In all the study groups, we found negative correlations between perceived stress and sleep quality; this finding is supported by previous reports, where perceived stress or academic stress correlated inversely with sleep quality [37,38,39]. In women, we identified a positive correlation between perceived stress and illicit substance use. This finding aligns with models that position stress as a precipitant of substance-use behavior [40]. Additionally, in both the bivariate and multivariable analyses of the full sample, perceived stress was inversely associated with age. This pattern accords with evidence from the COVID-19 pandemic showing that older adults reported lower pandemic-related stress than younger individuals [41] and with reports finding that stress tends to diminish in the transition from adulthood into midlife with substantial variability [42]. Additionally, in the multivariable analysis of the total sample, there was a non-significant trend toward a positive correlation with morbidity count. This aligns with our group’s earlier findings of a positive correlation between disease burden and academic stress [38]. Other sociodemographic variables—including daily physical activity and monthly earnings—showed inverse associations with perceived stress in the bivariate and/or multivariable analyses. These findings are consistent with prior evidence; for example, in a large cohort of Korean adults, higher exercise levels were associated with lower psychological stress [43]. Moreover, Cundiff et al. [44] reported that individuals with lower income experienced greater psychological stress. In addition, the positive correlation between being employed and psychological stress contrasts with previous reports showing an inverse association between these variables [45,46]. However, our findings suggest that higher work responsibilities could be related to higher perceived stress in this sample. All these correlations suggest that lifestyle factors are intrinsically correlated with perceived stress and should be considered when correlation analyses are intended.

With respect to biochemical correlates, perceived stress was positively associated with platelet count in bivariate analyses of the total sample and the women’s subgroup and in the multivariable model for the total sample. This pattern is consistent with stress-related platelet activation. Previous studies have shown that mental stress can increase pro-inflammatory platelet markers [47], as well as platelet-secreted proteins [48]. However, to our knowledge, no prior study has examined the association between perceived stress and platelet count.

Regarding the negative correlations reported between perceived stress and serum lipids, although some reports have documented a positive association between serum lipids (either total cholesterol, LDL, or triglycerides) with depressive symptoms or psychological discomfort [49,50], a large study that performed a Mendelian randomization analysis [51] found that while triglycerides increased depressive symptoms, LDL-cholesterol was inversely related to self-harm and suicide. In line with these results, a recent review reported that lower TC and other lipid levels are associated with suicidality and violent behavior [52]. Although no clear sex differences in suicidality were identified, this observation aligns with our results and suggests that lower lipid levels may be linked to poorer mental health outcomes, including stress and depressive symptoms. In this sense, it is interesting that the correlations observed with depressive symptoms were higher than those observed with stress symptoms, consistent with some previous reports [50,51]. These correlations have been related with the possibility that reduced levels of serum cholesterol may be accompanied by changes in the function and number of serotonin receptors and by a diminishment of serotonin entry into brain cells [53]. Altogether, these results suggest that there is an association between perceived stress and lipid levels. However, more studies are needed to determine the types of lipids associated with the psychological variables, as well as the influence of sex and other confounding factors in these associations.

In relation to hematologic indices, leukocyte count showed a positive bivariate correlation with perceived stress in the total sample and a non-significant positive trend in men; however, this association did not persist in the multivariable models for either subgroup or the overall sample. These findings align with prior evidence: Maes et al. reported exam-related stress in university students to be accompanied by shifts in leukocyte subset distribution, with significant positive relationships between stress-induced changes in perceived stress and both total leukocyte counts and specific white-cell subsets [54]. Additionally, in a male Sprague–Dawley rat model, Dhabhar et al. reported that stress-induced norepinephrine (NE) increased total leukocyte counts [55]. Regarding erythrocytes, our multivariable analysis revealed a significant positive association in the male subgroup. We did not identify published studies directly linking red blood cell count with perceived stress. However, a previous report described stress-related alterations in erythrocyte parameters, including increased hemoglobin, hematocrit, mean corpuscular volume, and other red cell indices, during periods of heightened perceived stress in students undergoing academic stress [54].

With respect to molecular markers, a marginal inverse association between perceived stress and 8-hydroxy-2′-deoxyguanosine (8-OHdG) was observed in bivariate analyses among women; however, 8-OHdG did not enter any of the multivariable models. Instead, IL-1β showed a marginal positive association with perceived stress in the total sample. This finding is consistent with prior evidence reporting moderate positive correlations between subjective stress and IL-1β in healthy male volunteers [56]. Although IL-1β was the only inflammatory marker associated with stress in our data, its positive relationship with perceived stress is biologically plausible given stress-related activation of the HPA axis and subsequent neuroendocrine–immune changes that promote chronic inflammation [57]. In the multivariable models, 8-isoprostane—a marker of lipid peroxidation and oxidative stress—was positively associated with perceived stress in women but inversely associated in men. These findings coincide with a report showing that 8-iso-prostaglandin-F2α (IsoP), a subtype of 8-isoprostane, was positively associated with anticipatory cortisol reactivity in chronically stressed caregivers, while in the control group of less-stressed individuals, higher stress scores were negatively correlated with IsoP levels [58]. These findings may help explain the sex differences observed in our study, where 8-isoprostane was positively associated with perceived stress among women—who reported higher stress levels than men—but was inversely associated with perceived stress among men. Altogether, these observations coincide with reports showing none to low correlations between perceived stress and immunological markers in the bivariate analyses but significant or higher positive correlations in the multivariable analyses [19,59]. However, it is important to note that most studies included different biomarkers, and none of the analyzed studies performed sex-segmented analyses, which could have modified the results.

The final biomarker associated with perceived stress was global DNA methylation, where it was inversely associated with PSS-10 in the women’s multivariable model but positively associated in the men’s model. These results are consistent with epigenome-wide evidence linking psychosocial stress to DNA methylation alterations [20], as well as with gene-specific methylation associated with anxiety and depression [60]. They also align with our prior report showing an inverse association between perceived stress and global DNA methylation in women [61]. The difference in the direction of the association between perceived stress and global DNA methylation between men and women may be explained by the fact that men generally exhibit higher baseline methylation levels, leading to opposite slopes in response to the same stimulus [61]. These differences may also be related to the influence of inflammation and oxidative stress on DNMT activity [62,63]; however, this relationship could be different in each sex and could be related to hypomethylation in women and hypermethylation in men. However, only experimental studies can determine a possible causality in this relationship and possible sex-related differences.

This study has several limitations. First, the sample size is modest, which increases random error and reduces the precision of estimated correlations—particularly given the number of comparisons and the multivariable models considered. Accordingly, the findings should be interpreted cautiously and viewed as preliminary, providing a basis for larger confirmatory studies. Second, the cross-sectional design precludes inferences about causality. Nonetheless, the inclusion of a broad set of covariates helps mitigate confounding through statistical adjustment, and sex-stratified analyses allowed for the identification of associations that may be specific to women or men. Third, the sample was not randomly selected, which limited the generalization of the results, as individuals who provided consent and chose to participate may differ from the broader non-participating population. Finally, the inclusion of only some serum lipids (TC and triglycerides), and the lack of inclusion of other cholesterol subtypes, limited us in distinguishing the potential differential correlations between these lipids and perceived stress.

## 5. Conclusions

In conclusion, perceived stress was significantly higher in women than in men and was associated with multiple personal, biochemical variables and molecular biomarkers in both sexes, with more significant correlations in women. The variable most constantly correlated (negatively) with perceived stress was sleep quality, while the lipid levels (cholesterol and triglycerides) showed negative correlations with perceived stress; conversely, platelets and erythrocytes showed positive correlations with stress. Regarding the molecular biomarkers, it was interesting that all of them showed significant correlations only in the multivariable analyses; in this sense, it is noteworthy that IL-1β was the only inflammatory cytokine associated with stress, while 8-isoprostane was significantly correlated with stress in the multivariable analyses of both sexes but in opposite directions in each sex. Likewise, the opposite correlations between stress and global DNA methylation in each sex in the multivariable analyses suggest that psychological and biological variables may influence the correlations between stress and epigenetics. The difference observed in the studied associations between sexes can be attributable to the higher levels of perceived stress observed in women than in men, along with other biological factors, such as hormonal ones. Likewise, the differences in the relationships observed between global DNA methylation and perceived stress in both sexes can be explained by the different factors associated with global DNA methylation in each sex. Further larger and longitudinal studies including the studied variables, as well as gene-specific methylation studies—including genes such as DNMT, transcription factors related to immune response, and those related to oxidative stress—will clarify the relationship between psychological stress, biochemical variables, and molecular biomarkers.

## Figures and Tables

**Table 1:** Comparison of studied variables between sexes.

Variables	Total *n* = 157	Female *n* = 83	Male *n* = 74	*p*
**Sociodemographic**				
Age (years), median (range)	24.0 (18–58)	23.0 (18–58)	25.5 (18–54)	0.535
Highest education level, *n* (%)				
Middle school	7 (4.5)	4 (4.8)	3 (4.1)	**0.019**
High school	66 (42.0)	41 (49.4)	25 (33.8)	
Bachelor’s degree	66 (42.0)	35 (42.2)	31 (41.8)	
Master’s degree	11 (7.0)	2 (2.4)	9 (12.2)	
Doctorate (Ph.D.)	7 (4.5)	1 (1.2)	6 (8.1)	
With a romantic partner, *n* (%)	88 (56.1)	42 (50.6)	46 (62.2)	0.145
Having children, *n* (%)	44 (28.0)	22 (26.5)	22 (29.7)	0.653
Employed, *n* (%)	105 (66.9)	52 (62.7)	53 (71.6)	0.233
Monthly earnings (MXN), *n* (%)				
Low	8 (5.1)	7 (8.4)	1 (1.4)	**<0.001**
Lower-middle	48 (30.6)	33 (39.8)	15 (20.3)	
Upper-middle	61 (38.8)	33 (39.8)	28 (37.7)	
High	25 (15.9)	6 (7.2)	19 (25.7)	
Very high	15 (9.6)	4 (4.8)	11 (14.9)	
Free time (hours), median (range)	4.0 (0.0–12.0)	4.0 (0–12.0)	4.0 (0–12.0)	0.077
Daily exercise (hours), *n* (%)				
0 to 0.5	64 (40.7)	35 (42.2)	29 (39.2)	0.476
1.0 to 1.5	59 (37.6)	34 (41.0)	25 (33.8)	
2.0 to 2.5	24 (15.3)	10 (12.0)	14 (18.9)	
>3	10 (6.4)	4 (4.8)	6 (8.1)	
Weekly dietary supplement use, *n* (%)				
Never	87 (55.5)	41 (49.4)	46 (62.1)	0.161
Less than once	31 (19.7)	16 (19.3)	15 (20.3)	
1 to 2	12 (7.6)	8 (9.6)	4 (5.4)	
3 to 4	12 (7.6)	6 (7.2)	6 (8.1)	
Daily	15 (9.6)	12 (14.5)	3 (4.1)	
Morbidity count, median (range)	2.0 (0–10)	3.0 (0–10)	2.0 (0–8)	**0.003**
Daily intake of drugs	0.0 (0.0–4.0)	0.0 (0.0–4.0)	0.0 (0.0–3.0)	0.081
Sleep quality, median (range)	3.0 (0.0–4.0)	2.8 (0.4–4.0)	3.2 (0.0–4.0)	0.122
Alcohol use frequency, *n* (%)				
Never	23 (14.6)	12 (14.5)	11 (14.9)	**0.009**
2 to 4 times per year	41 (26.1)	27 (32.5)	14 (18.9)	
Once a month or less	63 (40.2)	36 (43.4)	27 (36.4)	
2 to 3 times per week	27 (17.2)	6 (7.2)	21 (28.4)	
4 or more times per week	3 (1.9)	2 (2.4)	1 (1.4)	
Smoking frequency, *n* (%)				
Never	130 (82.8)	71 (85.6)	59 (79.6)	0.369
2 to 4 at year	8 (5.1)	5 (6.0)	3 (4.1)	
At least one monthly	5 (3.2)	2 (2.4)	3 (4.1)	
2 to 3 weeklies	5 (3.2)	3 (3.6)	2 (2.7)	
>4 weekly	9 (5.7)	2 (2.4)	7 (9.5)	
Consumption of the seven evaluated illicit substances, Mean ± SD	0.0 (0.0–07)	0.0 (0.0–0.3)	0.0 (0.0–0.7)	0.091
**Psychological**				
PSS-10, mean ± SD	1.5 ± 0.7	1.6 ± 0.8	1.4 ± 0.6	**0.048**
**Biochemicals**				
Leukocytes (1 × 10^3^/μL), median (range)	5.8 (3.4–11.0)	5.9 (3.4–11.0)	5.8 (3.5–8.5)	0.524
Lymphocytes (1 × 10^3^/μL), median (range)	1.8 (0.8–3.5)	1.7 (0.9–3.0)	1.8 (0.8–3.5)	0.456
Monocytes (1 × 10^3^/μL), median (range)	0.2 (0.1–0.4)	0.2 (0.1–0.4)	0.2 (0.1–0.4)	0.105
Granulocytes (1 × 10^3^/μL), median (range)	3.8 (1.9–7.9)	3.9 (2.0–7.9)	3.8 (1.9–6.3)	0.187
Platelets (1 × 10^3^/μL), mean ± SD	223.7 ± 47.0	236.5 ± 48.9	209.4 ± 40.7	**<0.001**
Hemoglobin (g/dL), median (range)	13.4 (7.3–19.2)	12.7 (7.3–17.4)	14.5 (12.9–19.2)	**<0.001**
Triglycerides (mg/dL), median (range)	97.8 (21.7–625.8)	97.6 (23.7–357.9)	99.6 (21.7–625.8)	0.256
Total cholesterol (mg/dL), mean ± SD	185.1 ± 38.1	180.8 ± 33.7	189.9 ± 42.1	0.140
Glucose (mg/dL), median (range)	88.5 (59.4–232.0)	83.8 (59.4–120.6)	92.3 (61.6–232.0)	**<0.001**
Urea (mg/dL), median (range)	27.0 (10.0–143.7)	26.2 (14.2–143.7)	27.0 (10.0–58.7)	0.306
Global DNA methylation (%), median (range)	0.4 (0.0–2.1)	0.4 (0.0–1.9)	0.5 (0.1–2.1)	**0.045**
**Levels of inflammatory and oxidative stress biomarkers**				
TNF-α (pg/mL), median (range)	177.2 (15.6–1000.0)	62.9 (15.6–1000.0)	403.5 (15.6–1000.0)	0.132
IL-8 (pg/mL), median (range)	9.5 (7.8–462.0)	9.8 (7.8–462.0)	9.4 (7.8–380.5)	0.259
IL-6 (pg/mL), median (range)	5.0 (3.1–329.0)	6.2 (3.1–329.0)	4.2 (3.1–23.3)	**0.006**
IL-1β (pg/mL), median (range)	13.4(7.8–1000.0)	13.7 (7.8–1000.0)	13.3 (8.3–27.4)	0.410
IL-10 (pg/mL), median (range)	61.1 (7.8–1741.6)	43.3 (7.8–1741.6)	105.8 (7.8–1741.6)	0.399
8-Isoprostane (pg/mL), median (range)	296.8 (230.1–418.3)	291.0 (230.1–418.3)	300.9 (246.4–364.4)	0.175
8-OHdG, median (range)	1.9 (0.6–10.0)	1.9 (0.6–10.0)	2.0 (1.1–2.9)	0.218
**Anthropometrics and blood pressure**				
BMI, median (range)	25.6 (16.4–39.9)	25.9 (16.4–39.9)	25.2 (18.7–38.9)	0.783
WHR, median (range)	0.8 (0.7–1.2)	0.8 (0.7–1.0)	0.9 (0.7–1.2)	**<0.001**
Systolic BP (mmHg), median (range)	112.0 (80.0–164.0)	106.0 (80.0–155.0)	120.0 (90.0–164.0)	**<0.001**
Diastolic BP (mmHg), median (range)	77.0 (57.0–120.0)	75.0 (57.0–106.0)	79.0 (62.0–120.0)	**0.025**

The names of the group of variables and the significant *p* values are highlighted in bold. Quantitative variables are expressed in median and range and in mean ± standard deviation (SD). Qualitative variables are expressed in frequency and percentage. Comparisons between sexes were performed with chi-squared and Fisher’s exact tests for qualitative variables and with the *t*-test and Mann–Whitney U test for quantitative variables. Statistically significant values (*p* < 0.05). Monthly earnings (MXN: Mexican pesos): 5 categories, from low to very high; smoking and alcohol consumption frequency were measured, from 0 to 4 (never to more than 4 times in the week); perceived stress scale (PSS-10), from 0 to 3 (never to frequently); TNF-α: tumor necrosis factor alpha; IL: interleukin; 8-OHdG: 8-hydroxy-2-deoxyguanosine; BMI: body mass index, WHR: waist-to-hip ratio; and BP: blood pressure.

**Table 2:** Significant bivariate correlations on the total and sex-stratified samples between the studied variables and perceived stress.

Variables	Total Sample *n* = 157	Women *n* = 83	Men *n* = 74
Age	−0.283 **	−0.314 **	—
Monthly earnings	−0.193 *	—	−0.277 *
Illicit substance use	—	0.250 *	—
Morbidity count	0.190 *	0.215 ^†^	—
Sleep quality	−0.476 **	−0.509 **	−0.384 **
Leukocytes	0.159 *	—	0.223 ^†^
Platelets	0.175 *	0.245 *	—
Triglycerides	−0.163 *	—	−0.237 *
Total cholesterol	−0.245 **	−0.299 **	—
8-hidroxyguanosine	—	−0.186 ^†^	—
WHR	−0.184 *	—	—

* *p* < 0.05, ** *p* < 0.01. Correlations were performed with the Pearson or Spearman correlation tests. ^†^ This variable shows a marginal level of statistical significance, between 0.05 and 0.1. WHR: waist-to-hip ratio.

**Table 3:** Multivariable regression analysis for perceived stress in overall sample and stratified by sex.

Variables	B	Beta Coefficient	Significance	Change in R^2^	Tolerance
**Total sample**					
Constant	2.642	—	<0.001	—	—
Sleep quality	−0.297	−0.399	<0.001	0.223	0.878
Total cholesterol	−0.003	−0.158	0.041	0.026	0.756
Employed	0.401	0.267	<0.001	0.018	0.740
Age	−0.015	−0.231	0.007	0.017	0.630
Daily exercise	−0.137	−0.171	0.018	0.024	0.865
Platelets	0.002	0.129	0.058	0.017	0.979
IL-1β	0.001	0.126	0.066	0.013	0.968
Morbidity count	0.040	0.117	0.096	0.012	0.914
**Women**					
Constant	2.399	—	0.003	—	—
Sleep quality	−0.408	−0.503	<0.001	0.256	0.947
Total cholesterol	−0.005	−0.216	0.021	0.058	0.956
Global DNA methylation	−0.630	−0.212	0.021	0.035	0.987
8-isoprostane	0.005	0.213	0.023	0.043	0.948
**Men**					
Constant	2.017	—	0.098	—	—
Sleep quality	−0.226	−0.345	<0.001	0.170	0.950
Global DNA methylation	0.537	0.254	0.018	0.028	0.778
8-isoprostane	−0.004	−0.177	0.068	0.040	0.949
Monthly earnings	−0.193	−0.310	0.002	0.041	0.919
Diastolic BP	0.015	0.243	0.021	0.046	0.806
WHR	−1.761	−0.241	0.026	0.040	0.768
Erythrocytes	0.391	0.213	0.031	0.030	0.913
Daily exercise	−0.117	−0.178	0.083	0.026	0.831

The names of the analyzed samples are highlighted in bold. R of the models: total: 0.592, women: 0.626, and men: 0.668.

## Data Availability

The datasets presented in this article are not readily available because the data are part of an ongoing study or due to technical/time limitations. The datasets produced in this study, together with the corresponding statistical analyses, can be obtained from the authors upon request at aniel.brambila@academicos.udg.mx.

## Supplementary Materials

The following supporting information can be downloaded at https://www.mdpi.com/article/10.3390/biom16030405/s1: Table S1: Self-reported morbidity over the previous six months covering 29 conditions.
