# Supplementary material for: Association Between Biochemical, Inflammatory, Oxidative Stress and DNA Methylation Biomarkers with Perceived Stress in Mexican Individuals
Source: Biomolecules. 2026 Mar 10;16(3):405. doi: 10.3390/biom16030405 (PMC13024258; doi:10.3390/biom16030405)
Supplement: Supplementary file 1 [file biomolecules-16-00405-s001.zip › biomolecules-4145141-supplementary.pdf]

Table S1. Self-reported morbidity over the previous six months and moribidity conditions detected during the study.

Disease, n (%)	Women n=83	Men n=74
1.- Hypertension	2 (2.4)	0 (0.0)
2.- Diabetes mellitus (type 1 or 2)	2 (2.4)	2 (2.7)
3.- Thyroid disease	5 (6.0)	0 (0.0)
4.- Allergies (e.g., asthma, conjunctivitis)	19 (22.9)	13 (17.6)
5.- Dyslipidemia	12 (14.5)	5 (6.8)
6.- Gastritis	34 (41.0)	22 (29.7)
7.- Colitis/irritable bowel syndrome	34 (41.0)	17 (23.0)
8.- Migraine or tension headache	44 (53.0)	22 (29.7)
9.- Dermatologic problems (acne, neurodermatitis)	30 (36.1)	21 (28.4)
10.- Gastrointestinal infections	25 (30.1)	20 (27.0)
11.- Peptic ulcer disease	2 (2.4)	5 (6.8)
12.- Sinusitis	9 (10.8)	11 (14.9)
13.- kidney disease (renal failure, nephrolithiasis)	3 (3.6)	0 (0.0)
14.- Anorexia/bulimia	3 (3.6)	0 (0.0)
15.- Depression requiring medication	6 (7.2)	1 (1.4)
16.- Anxiety requiring medication	10 (12.0)	2 (2.7)
17.- Myocardial infarction/angina	1 (1.2)	1 (1.4)
18.- Rheumatic diseases (rheumatoid arthritis, lupus, ankylosing spondylitis)	0 (0.0)	4 (5.4)
19.- Heart failure	0 (0.0)	0 (0.0)
20.- Stroke/cerebral infarction	0 (0.0)	0 (0.0)
21.- Chronic infections (HIV, tuberculosis, long COVID, etc.)	1 (1.2)	2 (2.7)
22.- Cancer (breast, cervical, prostate, skin)	0 (0.0)	0 (0.0)
23.- Leukemia/lymphoma	0 (0.0)	0 (0.0)
24.- Advanced cancer (metastatic)	0 (0.0)	0 (0.0)
25.- Venous disease (venous insufficiency, varicosities)	14 (16.9)	1 (1.4)
26.- Liver disease (hepatitis, cirrhosis, fatty liver)	3 (3.6)	1 (1.4)
27.- Chronic lung disease	0 (0.0)	0 (0.0)
28.- Respiratory infections (including COVID)	14 (16.9)	14 (18.9)
29.- Hemiplegia (paralysis)	0 (0.0)	0 (0.0)
Any other chronic illness requiring ongoing treatment	7 (8.4)	3 (4.1)
